# Toll-like receptor 2 and type 2 diabetes

**DOI:** 10.1186/s11658-016-0002-4

**Published:** 2016-07-28

**Authors:** Zahra Sepehri, Zohre Kiani, Ali Akbar Nasiri, Farhad Kohan

**Affiliations:** 1 Department of Internal Medicine, Zabol University of Medical Sciences, Zabol, Iran; 2 Zabol Medicinal Plant Research Center, Zabol University of Medical Sciences, Zabol, Iran; 3 Department of Medicine, Kerman University of Medical Sciences, Kerman, Iran; 4 Department of Internal Anesthesiology, Zabol University of Medical Sciences, Zabol, Iran; 5 General Physician, Zabol University of Medical Sciences, Zabol, Iran

**Keywords:** Type 2 diabetes, TLR2, Complication, Innate immunity, DAMP, PAMP, MYD88, Activator protein 1, NF-kB, Leucine-rich repeats

## Abstract

Innate immunity plays a crucial role in the pathogenesis of type 2 diabetes and related complications. Since the toll-like receptors (TLRs) are central to innate immunity, it appears that they are important participants in the development and pathogenesis of the disease. Previous investigations demonstrated that TLR2 homodimers and TLR2 heterodimers with TLR1 or TLR6 activate innate immunity upon recognition of damage-associated molecular patterns (DAMPs). Several DAMPs are released during type 2 diabetes, so it may be hypothesized that TLR2 is significantly involved in its progression. Here, we review recent data on the important roles and status of TLR2 in type 2 diabetes and related complications.

## Background

Type 2 diabetes, also referred to as adult-onset diabetes or as non-insulin-dependent diabetes mellitus (NIDDM) is increasingly common [1, 2]. The heightened levels of blood glucose are associated with several complications, including nerve damage (neuropathy), which is caused by injury to the walls of the capillaries that nourish the nerves and results in tingling, numbness, burning or pain [3, 4]. Nephropathy is also associated with injuries to the glomeruli [5]. Eye damage or retinopathy is induced by glucose-related damage to the blood vessels of the retina [6–8]. Type 2 diabetes correlates significantly with cardiovascular disease, including heart attack, angina, stroke and atherosclerosis. Moreover, investigations have shown a link between type 2 diabetes and brain diseases, such as mild cognitive impairment, Alzheimer’s disease and vascular dementia [9]. Other complications include periodontitis [10, 11], cystic fibrosis [12], hypertension [13] and hearing impairment [14].

The parameters of innate immunity are associated with type 2 diabetes and its complications [15, 16]. Therefore, it has been hypothesized that the disease is immune dependent [16, 17]. For instance, in cases of type 2 diabetes, immune cells produce inappropriate levels of inflammatory cytokines, which may have a deleterious effect on the pathogenesis of type 2 diabetes [18]. Elevated serum levels of innate immunity inflammatory cytokines such as IL-6 [19], IL-18 [20] and TNF-α [19] have been reported for subjects with type 2 diabetes and related complications.

Several cross-sectional studies also confirmed the relationship between innate immunity and type 2 diabetes. For instance, studies were made of non-diabetic subjects and patients with type 2 diabetes determined as impaired glucose tolerance or impaired fasting glucose. They identified that innate immunity soluble molecules, such as C-reactive protein (CRP) and its related cytokines, are: positively associated with insulin resistance, plasma insulin concentration, circulating triglyceride level, and BMI and waist circumference measurements; and negatively correlated with HDL concentration [21]. Additionally, it has been documented that exercise can improve type 2 diabetes complications through its immunomodulatory effects [22].

**Fig. 1:**
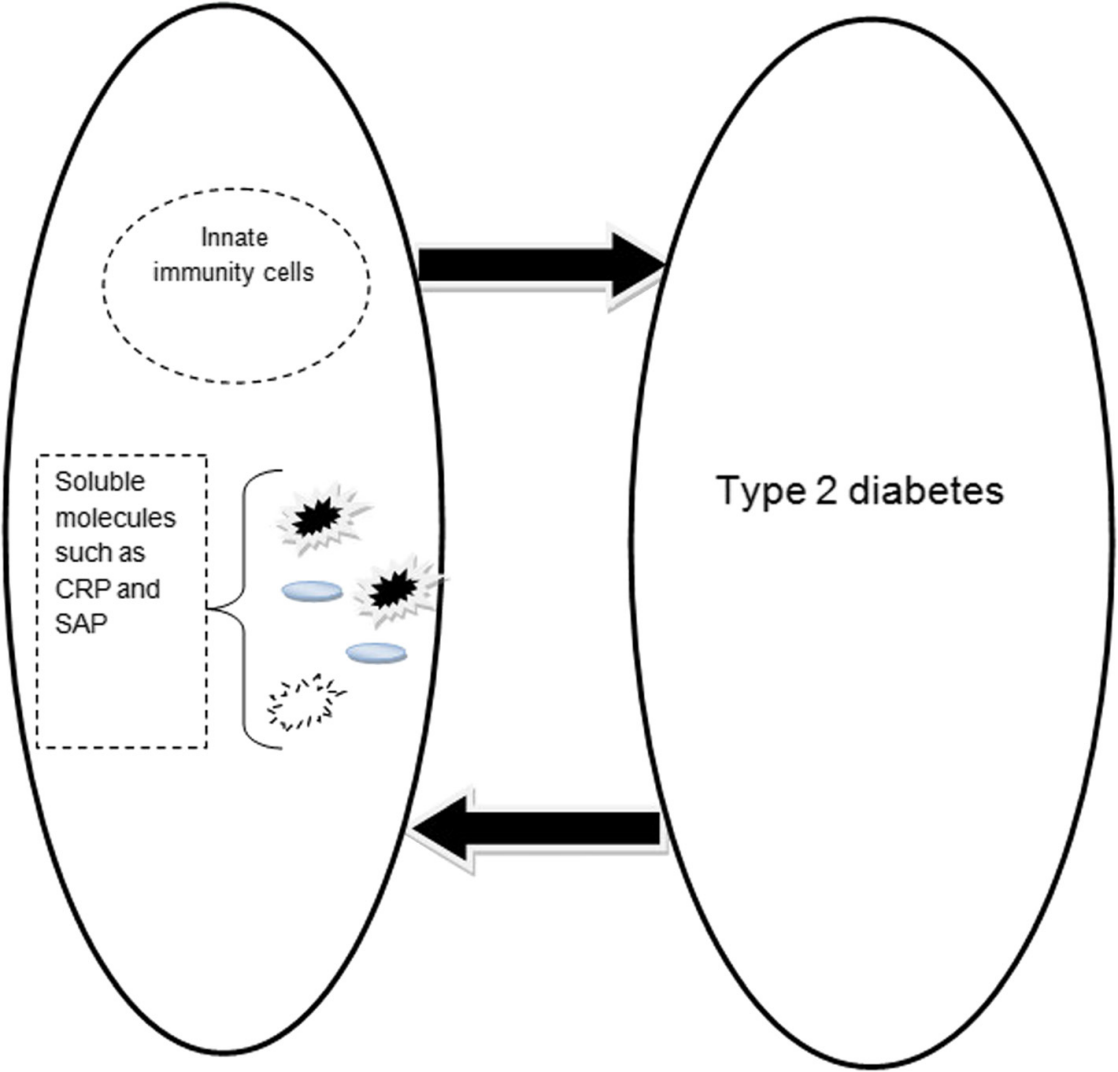
There is a mutual avenue between type 2 diabetes and innate immunity. Type 2 diabetes can activate innate immunity and its pathogenesis can be accelerated by activated innate immunity. CRP: C-reactive protein, SAP: Serum amyloid protein

Although a significant relationship between innate immunity and type 2 diabetes has been confirmed, it has yet to be clarified if innate immunity activation is a major mechanism in the induction of type 2 diabetes or if the disease activates innate immunity. It seems that it is mutual (Fig. 1), so evaluation of innate immunity receptors and their signaling molecules should our knowledge of the condition and relationship.

Toll-like receptors (TLRs) are the innate immune cell receptors. They play pivotal roles in the recognition of damage-associated molecular patterns (DAMPs), which occur during type 2 diabetes. Their recognition leads to several functions of innate immune cells, including phagocytosis [23], cytokine production [24], and expression of co-stimulatory molecules [25] and adhesion molecules [26, 27].

Toll-like receptor 2 (TLR2) plays significant roles in the induction of innate immune cells through a MYD88-dependent pathway [28, 29]. Interestingly, there is some evidence that confirms the suppressive roles of TLR2 and ligand interactions on immune responses [30]. Therefore, TLR2 may participate in the development and pathogenesis of immune-related diseases, including type 2 diabetes. Accordingly, the altered expression and functions of TLR2 and its intracellular molecular signaling may be associated with the mechanisms that result in the progression and pathogenesis of type 2 diabetes and its related complications.

### Toll-Like Receptor 2

TLR2 (TIL4, CD282) was identified and characterized in 1998 [31]. It is an innate immune cell receptor that recognizes several pathogen-associated molecular patterns (PAMPs) and DAMPs and subsequently activates MYD88-dependent intracellular signaling [29]. Chromosome 4 (4p32) is the location of the TLR2 gene. This molecule is a type I transmembrane protein that has a similar structure to other TLRs, consisting of the following domains from N-terminal to C-terminal: extracellular leucine-rich repeat (LRR) domains; a transmembrane domain; and a toll/interleukin-1 receptor (TIR) domain. TLR2 is expressed in several immune cell types, including macrophages and dendritic cells, and non-immune cell types, including endothelial cells, epithelial cell lines and hepatocytes [32, 33].

TLR2 recognizes its ligands in both homodimer and heterodimer (with TLR1 or 6) forms [34]. In its homodimer form, it recognizes lipopolysacharide (LPS), porins, lipoprotein, lipoteichoic acid, bacterial peptidoglycan, viral hemagglutinin and glycoproteins. In its TLR2/1 heterodimer form, it recognizes bacterial triacylated lipopeptides and synthetic triacylated lipopeptide (Pam3CSK4) [35], and in its TLR2/6 heterodimer, it recognizes bacterial diacylated lipopeptides and lipoteichoic acid [36]. As mentioned previously, TLR2 also recognizes some DAMPs as endogenous ligands, including human glycosaminoglycan hyaluronan [37], β-defensin-3, heat shock proteins and high mobility group box 1 protein, some of which are released during inflammatory diseases like type 2 diabetes [30, 38–40].

**Fig. 2:**
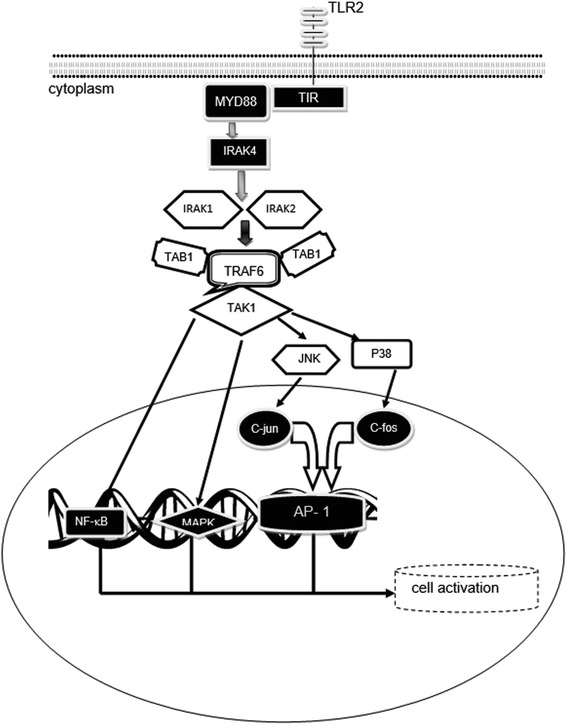
Following TLR2-ligand interaction a signaling pathway is started using MYD88 as an adaptor molecule. The signaling pathway leads to the activation of several transcription factors, including NF-kB, MAPK and AP-1, and subsequently cell activation. Adapted from Bagheri et al. [29]

TLR2–ligand interactions lead to the activation of MAPK and MYD88-dependent signaling pathways (Fig. 2) [41, 42]. Although MYD88-dependent signaling pathway activation results in the phosphorylation and activation of pro-inflammatory transcription factors, such as IRF3, IRF7, AP-1 and NF-kB, it also induces the PI3K/AKT pathway, which leads to upregulation of IL-10, and activates SOCS proteins [43]. IL-10 is a major anti-inflammatory cytokine [44]. SOCS proteins also suppress MAPK and JAK–STAT signaling pathways [45]. Therefore, it appears that TLR2-ligand interaction leads to either activation or suppression of immune responses [46, 47].

The principal mechanisms causing the activation or suppression of immune responses by TLR2 are unclear, but it has been hypothesized that the TLR2 ligand concentration may be the determining factor.

### Type 2 Diabetes And TLR2

DAMPs are endogenous molecules that are produced and released by several cell systems during inflammation or infection [48, 49]. They can also be released during type 2 diabetes [50]. Both can be recognized by TLR2, leading to the either activation or suppression of immune cells. It has been documented that inflammation is a major cause of pancreatic beta cell dysfunction in type 2 diabetes [51, 52]. Therefore, the inflammatory effects of TLR2-ligand interaction may be an important factor in type 2 diabetes progression. Nackiewicz et al. showed that interaction between TLR2/6 and its related ligands results in the activation of macrophages and the production of IL-1 and IL-6 as pro-inflammatory cytokines that contribute to islet inflammation [51].

Several studies confirmed the important roles played by reactive oxygen species (ROS) in the pathogenesis of type 2 diabetes. Interestingly, activation of TLR2 by zymosan leads to ROS production by neutrophils in a manner dependent on TLR2 NADPH oxidase but not dependent on MAPK [53]. Hyperglycemia and chronic periodontitis also lead to upregulation of TLR2 in the gingival tissue of type 2 diabetes patients [54]. Interestingly, it has been demonstrated that insulin suppresses the expression of TLR2 at the mRNA level, possibly via downregulation of PU.1 [55]. Kuzmicki et al. revealed that the gestational diabetes patients had significantly higher TLR2 expression than pregnant women with normal glucose tolerance [56]. Interestingly, TLR2 was found to be upregulated in women who exhibited normal glucose tolerance but later developed gestational diabetes when compared to the women who remained normoglycemic [56]. Ahmad et al. demonstrated that the expression levels of TLR2 were upregulated in obese individuals [57]. Additionally, they showed that obese type 2 diabetes patients had higher expressions of TLR2 in comparison to obese patients without type 2 diabetes [57]. The phagocytic cells of type 2 diabetes patients have also upregulated TLR2 [58].

Another study also identified that TLR2 not only participates in the development of type 2 diabetes but is also involved in the pathogenesis of related vascular complications [59]. Moreover, via upregulation of IL-6 and osteopontin, TLR2 causes impaired insulin-mediated brain activities, which are an early step in the development toward type 2 diabetes [60]. Duarte et al. revealed higher mRNA levels of TLR2 in gingival biopsies from type 2 diabetes patients with chronic periodontitis in comparison to periodontally healthy patients [61]. Thus, it appears that the upregulation of TLR2 is a marker of type 2 diabetes rather than a marker of periodontitis. Rojo-Botello et al. also confirmed this result [62]. Another study identified that free fatty acids and high glucose levels upregulate the expression of TLR2 and TLR6, which resulted in increased activity of monocytes and increased production of superoxides, which are released in an NF-kB-dependent manner [63]. Ehses et al. also reported that a high-fat diet was unable to induce insulin resistance and beta cell dysfunction in TLR2-deficient mice [64]. Free fatty acids also play important roles in the induction of inflammation in pancreatic beta cells via TLR2 [65]. Interestingly, another study showed that not only TLR2 has been more highly expressed on the immune cells of type 2 diabetes patients than on those of healthy subjects, but also the levels of TLR2 ligands, including hyaluronan, HSP60, HSP70, HMGB1 and endotoxin, were higher [66]. TLR2 inhibition using a TLR2 antisense oligonucleotide (ASON) leads to recovery of insulin sensitivity and signaling in muscle and white adipose tissue of mice that were fed a high-fat diet [67]. It has also been documented that oxidized LDL, which is produced during type 2 diabetes, induced expression of TLR2 in macrophages [68]. The expression of TLR2 on the monocytes of obese women is also higher [68].

TLR2 also recognizes PAMPs such as exogenous microbial ligands, so it may be hypothesized that microbial infections could be important factors in the development of type 2 diabetes and may also participate in the pathogenesis of the disease. Interestingly, a study by Ajuwon et al. revealed that peptidoglycan derived from *Staphylococcus aureus* resulted in elevated TLR2 expression on the 3 T3-L1 adipocytes cell lines [69]. Chen et al. identified that treatments with agents that improve glycemic control are associated with decreased expressions of TLR2 and its related intracellular signaling molecules [70]. Previous studies demonstrated that decreased methylation is associated with higher expression of the genes [71]. Accordingly, CpGs methylation in the promoter of the TLR2 gene was significantly decreased in type 2 diabetes patients in comparison to the levels for the controls [72].

As mentioned in the previous section, TLR2 is also able to suppress immune cells through unknown mechanisms. A few studies demonstrated that TLR2 expression decreased during type 2 diabetes. For instance, a study by Cortez-Espinosa et al. showed that the percentage of TLR2-positive monocytes decreased in type 2 diabetes patients with poor glycemic control when compared to patients with appropriate glycemic control [73]. Another study revealed that although type 2 diabetes patients had higher serum levels of IL-6, the mRNA levels of TLR2 were lower than in healthy subjects [74]. Interestingly, their results, which were obtained under in vitro conditions, demonstrate that high glucose levels lead to reduced expression of TLR2 [74]. High doses of insulin (100 IU/ml), such as those seen in cases of type 2 diabetes, also increased IL-6 and decreased TLR2 expression [74]. In an in vitro study, it was shown that TLR4 but not TLR2 interaction with corresponded ligands leads to pancreatic beta cell apoptosis [75]. S6K1 plays key roles in driving insulin resistance and the induction of type 2 diabetes [76]. Kim et al. found that upregulation of S6K1 causes a significant reduction in NF-kB and AP-1 activities, which are induced by TLR2–ligand interactions [77].

Since more than 90 % of studies reported that TLR2 is positively associated with type 2 diabetes and its complications, it seems that the inflammatory effects of TLR2 during the condition are predominant. Collectively, it appears that TLR2 plays remarkable roles in the development of type 2 diabetes and related complications. Therapeutic approaches involving modulation of the expression of TLR2 and related signaling molecules could be considered as novel approaches for the treatment of type 2 diabetes.

There have not been many studies on the variation of the TLR2 gene in type 2 diabetes are rare. Liu et al. reported on the low frequency of TLR2 Arg677Trp and Arg753Gln polymorphisms in type 2 diabetes patients in the Chinese Han population [78]. Another study showed that the TLR2 R753Q polymorphism was not associated with type 2 diabetes in Mexican population [79]. Further studies need to be done to find the relationship between genetic variations and the diseases.

Other TLRs like TLR3 and 4 also play key roles in the induction of this disease. We discussed the roles played by TLR3 in its development in our previous review [80]. Our research confirmed that TLR4 can also participate in its pathogenesis [81]. TLR3 and TLR4 use another signaling pathway, the TRIF-dependent pathway, which may be important in the development of type 2 diabetes. Thus, it seems that TLR2 is not a unique innate immunity receptor involved in the development of the disease.

## Conclusion

TLR2 plays crucial roles in the initiation and pathogenesis of type 2 diabetes and related complications. Downregulation of TLR2 can be considered as a novel approach for the treatment of these conditions. Of particular interest in such research are these points:

TLR2 is upregulated in several tissues that are affected by adult-onset type 2 diabetes and the gestational version of the disease.

1. TLR2 induces the production of several molecules including ROS and pro-inflammatory cytokines, which contribute to the worsening type 2 diabetes and related complications.
2. The expression of TLR2 positively correlates with elevated serum levels of free fatty acids and glucose as well as obesity.
3. Several exogenous and endogenous ligands induce the activation of immune cells and the dysfunction of pancreatic beta cells in a TLR2-initiated pathway-dependent manner.
4. Different genetic variations and methylation may play key roles in the upregulation of TLR2 in type 2 diabetes patients.
5. PAMPs participate in the activation of TLR2-initiated pathways, so it may be hypothesized that infection can be considered a crucial candidate for the development of type 2 diabetes and related complications.
6. Upregulation of insulin is an important mechanism that suppresses the expression of TLR2.

## Abbreviations

AP-1, activator protein 1; CRP, C-reactive protein; DAMPs, damage-associated molecular patterns; IRF, interferon regulatory factor; JAK-STAT, Janus kinase-signal transducer and activator of transcription; LDL, low-density lipoprotein; LRRs, leucine-rich repeats; MAPK, mitogen-activated protein kinase; MYD88, myeloid differentiation primary response; NADPH, nicotinamide adenine dinucleotide phosphate; NF-kB, nuclear factor kappa-light-chain-enhancer of activated B cells; NIDDM, non-insulin-dependent diabetes mellitus; PAMP, pathogen-associated molecular pattern; PBMC, peripheral blood mononuclear cell; ROS, reactive oxygen species; SOCS, suppressor of cytokine signaling proteins; TLR, toll-like receptor; TIR, toll/interleukin-1 receptor
